# Indirect Measurement of Electron Energy Relaxation Time at Room Temperature in Two-Dimensional Heterostructured Semiconductors

**DOI:** 10.3390/ma15093224

**Published:** 2022-04-29

**Authors:** Algirdas Sužiedėlis, Steponas Ašmontas, Jonas Gradauskas, Aurimas Čerškus, Maksimas Anbinderis

**Affiliations:** 1 Center for Physical Sciences and Technology, Savanorių ave. 231, 02300 Vilnius, Lithuania; steponas.asmontas@ftmc.lt (S.A.); jonas.gradauskas@ftmc.lt (J.G.); aurimas.cerskus@ftmc.lt (A.Č.); maksimas.anbinderis@ftmc.lt (M.A.); 2 Vilnius Gediminas Technical University, Saulėtekio ave. 11, 10223 Vilnius, Lithuania

**Keywords:** electron energy relaxation time, two-dimensional electron gas, quantum well, microwave diode, voltage sensitivity, I-V characteristic

## Abstract

Hot carriers are a critical issue in modern photovoltaics and miniaturized electronics. We present a study of hot electron energy relaxation in different two-dimensional electron gas (2DEG) structures and compare the measured values with regard to the dimensionality of the semiconductor formations. Asymmetrically necked structures containing different types of AlGaAs/GaAs single quantum wells, GaAs/InGaAs layers, or bulk highly and lowly doped GaAs formations were investigated. The research was performed in the dark and under white light illumination at room temperature. Electron energy relaxation time was estimated using two models of I-V characteristics analysis applied to a structure with *n-n^+^* junction and a model of voltage sensitivity dependence on microwave frequency. The best results were obtained using the latter model, showing that the electron energy relaxation time in a single quantum well structure (2DEG structure) is twice as long as that in the bulk semiconductor.

## 1. Introduction

The realm of two-dimensional materials was widened when graphene was rediscovered, segregated, and characterized by A. Geim and K. Novoselov [1]. To date, more than 150 different families of two-dimensional (2D) layered materials are known [2]. The most prominent families of 2D materials are transition metal dichalcogenides [3], hexagonal boron nitride [4], black phosphorus [5], perovskite nanosheets [6], 2D material-based heterostructures [7], and topological insulators [8]. The 2D materials have a wide palette of energy bandgaps, spanning from the terahertz and mid-infrared range in the case of graphene and black phosphorus, covering the visible spectrum in the transition metal dichalcogenides, and reaching the ultraviolet spectral region in hexagonal boron nitride [9]. A distinctive feature of these 2D materials is the possibility of tuning their bandgap by controlling the number of layers, heterostructuring, varying layer strain, chemical doping, alloying, intercalation, substrate engineering, and with an external electric field [9]. Topological insulators, the 2D crystalline structures grown using the molecular beam epitaxy technique, attracted recent attention because of their prospective application in quantum computing. The existence of non-Abelian quasiparticles in a matter state known as a quantum Hall liquid has been evidenced both theoretically and experimentally [10,11]. The non-Abelian anions can retain a “memory” of their position in the past that could be used in the development of ultra-fast quantum computing systems that do not require error correction [12]. The core of the experimental setup that evidenced the existence of the non-Abelian anions was an MBE-grown, selectively doped GaAs/AlGaAs heterostructure hosting electrons of extremely high mobility at liquid helium temperature [11]. However, the GaAs/AlGaAs quantum heterostructures are interesting not only from a fundamental viewpoint. These structures are useful as a source of efficiency increase in the hot carrier solar energy converters since the quantization phenomenon slows down the thermalization processes in the space charge layer of the quantum heterostructures and improves hot carrier transfer out of an absorber [13]. In this way, the extraction of hot carriers leads to higher values of photovoltage and photocurrent generated in solar cells containing quantum wells [14].

Experimental and theoretical studies of the hot carrier relaxation time in GaAs/AlGaAs heterostructures revealed it to be much longer than was expected from the study of electron–longitudinal optical phonon interaction in a bulk material [15,16,17]. Different reasons for the increased electron energy relaxation time have been suggested by different authors: (*a*) enhanced screening of electron–longitudinal optical phonon interactions in a two-dimensional electron gas; (*b*) reduction of the electron–phonon interactions in a quantum well due to the confinement of the lattice modes; (*c*) presence of non-equilibrium population of the longitudinal optical phonons [18]. However, the statement that the electron cooling rate depends upon the dimensionality of the semiconductor structure still seems to be controversial [19]. Some researchers report that the electron cooling rate in GaAs quantum structures depends on their dimensions and the dynamic screening of the electron–phonon interaction [15,20], while others prefer to conclude that the cooling rate depends on the existence of hot phonons, i.e., on the presence of a large population of non-equilibrium optical phonons [16,21]. The charge carrier cooling rate was experimentally investigated by studying photoluminescence (PL) decay using a time-correlated single-photon counting method [19], or by combining optical and electrical techniques [16]. However, optical methods of investigation of hot carrier phenomena require a set of parallel quantum wells since a single quantum well does not give a sufficiently strong signal. Therefore, hot electron energy relaxation was studied by measuring the electron noise temperature in single InGaAs quantum wells [22,23]. Note that the above-mentioned optical and electrical methods of electron relaxation analysis were used under non-equilibrium conditions. Parameters of hot electron relaxation closer to fundamental ones are obtained in the case when electrons are treated in equilibrium with a crystal lattice. The microwave (MW) mixing technique was used to measure electron energy relaxation time in single AlGaAs/GaAs modulation-doped quantum wells under quasi-equilibrium conditions [24,25]. These measurements were carried out at a liquid helium temperature.

When the operation of a device is based on hot electron phenomena in a strong electric field, the solution of phenomenological current density, heat balance, heat flow density, current continuity, and Poisson equations in an approximation of warm electrons gives the possibility to extract the electron energy relaxation time from the experimentally measured current–voltage (I-V) characteristic [26] or frequency dependence of the detected thermovoltage of hot carriers under the impact of microwave radiation [27]. However, investigations of electron energy relaxation have been performed in bulk GaAs using point-contact [26] and planar [27] designs of MW diodes. Two-dimensional semiconductor heterostructures play an important role not only in the miniaturization of modern electronic devices but also in the increase of their operational speed. The performance of microwave, millimeter-wave, and terahertz-range devices has been substantially improved when selectively doped semiconductor heterostructures are employed. In this paper, we present a study of hot electron energy relaxation time in various two-dimensional electron gas (2DEG) semiconductor structures and compare the obtained time values in the sense of the dimensionality of the investigated formations.

## 2. Theoretical Background, Materials, Samples, and Methods

Electron heating by an electric field of microwave radiation is a powerful tool to investigate the electron energy relaxation time [28]. The operation of MW diodes based on *l-h* (*n-n^+^* or *p-p^+^*) point contact was analyzed assuming that slightly heated carriers (the warm carrier approach) obey the Maxwell–Boltzmann distribution [29]. The expression of detected voltage as a function of absorbed microwave power was derived by considering the electron energy relaxation time and other characteristic parameters of the device, such as carrier density, Fermi level, lattice temperature, and ohmic contact diameter. However, that expression did not involve the frequency of microwave radiation. The voltage responsivity of the *n-n^+^* point contact MW detector was derived by solving phenomenological electron transport and Poisson equations [30]. The radiation frequency was included in the expression; other important parameters, such as electron energy and Maxwell relaxation times, contact radius, and electron density, were accounted for as well. An analogous expression of voltage responsivity *S_i_* was derived in the case of a planar, asymmetrically necked microwave diode [27], and later, it was adopted for the planar diode on the base of a selectively doped semiconductor structure [31] in the form of

(1) $$S_{i}=\frac{U_{d}}{P_{i}}=\frac{2R_{sh}\mu_{0}\tan\alpha }{3d^{2}\ln\frac{a}{d}}\frac{P}{P_{i}}N,$$

where *U_d_* is the voltage detected over the ends of the planar MW diode, $P_{i}$ marks the entire power incident on the diode, *P* stands for the MW power absorbed by the diode, $R_{sh}$ and $\mu_{0}$ note the sheet resistance of the selectively doped structure and electron mobility, respectively, *a* and *d* are the widths of the asymmetrically necked semiconductor formation in its widest and narrowest part, respectively, and *α* is the widening angle of the active part. The frequency-dependent factor *N* is expressed as

(2) $$\begin{array}{r} N=\left[\frac{1+{\left(\omega \tau_{M}\right)}^{2}}{{\left(\omega \tau_{M}\right)}^{2}}\left\{\tau_{\varepsilon }\left[1+\frac{s^{2}}{1+{\left(\omega \tau_{\varepsilon }\right)}^{2}}\right]\ln\left[1+{\left(\omega \tau_{M}\right)}^{2}\right]+\tau_{M}\left[\frac{3}{2}-\frac{s\left(1-s\right){\left(\omega \tau_{\varepsilon }\right)}^{2}}{1+{\left(\omega \tau_{\varepsilon }\right)}^{2}}\right]\times \right.\right.\\ \left.\left.\times \left[\frac{1}{\omega \tau_{M}}\arctan\left(\omega \tau_{M}\right)-\frac{1}{1+{\left(\omega \tau_{M}\right)}^{2}}\right]\right\}+\frac{s\left(1-s\right)\tau_{\varepsilon }}{1+{\left(\omega \tau_{\varepsilon }\right)}^{2}}\right]\frac{1}{1+{\left(\omega \tau_{i}\right)}^{2}} \end{array}$$

Here, *ω* denotes the angular frequency of microwave radiation, *s* is the exponent in the dependence of electron momentum relaxation time $\tau_{i}$ on electron energy *E*, $\tau_{\varepsilon }$ marks the electron energy relaxation time, and $\tau_{M}$ is the Maxwell relaxation time in the active layer. Thus, an approximation of the experimental dependence of the voltage responsivity on frequency to Equation (1) can be applied to evaluate the electron energy relaxation time and the share of incident microwave power (P/$P_{i}$) absorbed in the MW diode developed on the base of a two-dimensional semiconductor structure.

Investigation of the DC electrical properties of the asymmetrically shaped semiconductor structures also gives the possibility to obtain the electron energy relaxation time. However, this method requires perfect ohmic contacts to ensure that the physical phenomena under interest occur only in the bulk of a semiconductor. In the case of a perfect point-contact *n*^+^-*n-*Si junction, asymmetry of the I-V characteristic of the MW diode in the region of warm electrons can be expressed as [32]

(3) $$\Delta R=R_{b}-R_{f}=\frac{\left[\left(1+s\right)\tau_{\varepsilon }+\tau_{M}\right]U}{3\pi en_{0}r_{0}^{3}},$$

where $R_{b}$ and $R_{f}$ are the values of electrical resistance of the point-contact diode backward and forward-biased with voltage *U*, respectively, $n_{0}$ denotes the electron density in the *n*-region of the *n*^+^-*n*-junction, and $r_{0}$ marks the radius of the point contact. An analogous expression of the I-V asymmetry was derived for the planar MW diode on the base of the asymmetrically necked *n*^+^-*n*-junction [33],

(4) $$\Delta R=\frac{4U\rho \tan\alpha \left[\left(1+s\right)\tau_{\varepsilon }+\tau_{M}\right]\;\;}{3hd^{2}\ln\frac{a}{d}},$$

where *h* is the thickness of the semiconductor structure, *ρ* stands for the resistivity of its *n*-region, and *α* notes the widening angle of the *n*-region. Thus, Equation (4) allows us to calculate the electron energy relaxation time $\tau_{\varepsilon }$. Another option of estimating the electron energy relaxation time by using the I-V characteristic of a semiconductor structure with ohmic contacts was described in Ref. [34]. If the nonlinearity coefficient of the characteristic *β* is known, the electron energy relaxation time is found as [34]

(5) $$\tau_{\varepsilon }=-\frac{3}{2}\frac{kT\beta }{e\mu_{0}s},$$

where *k* is the Boltzmann constant, *T* notes the lattice temperature, and *e* and $\mu_{0}$ mark the elementary charge and low-field mobility, respectively.

**Figure A1:**
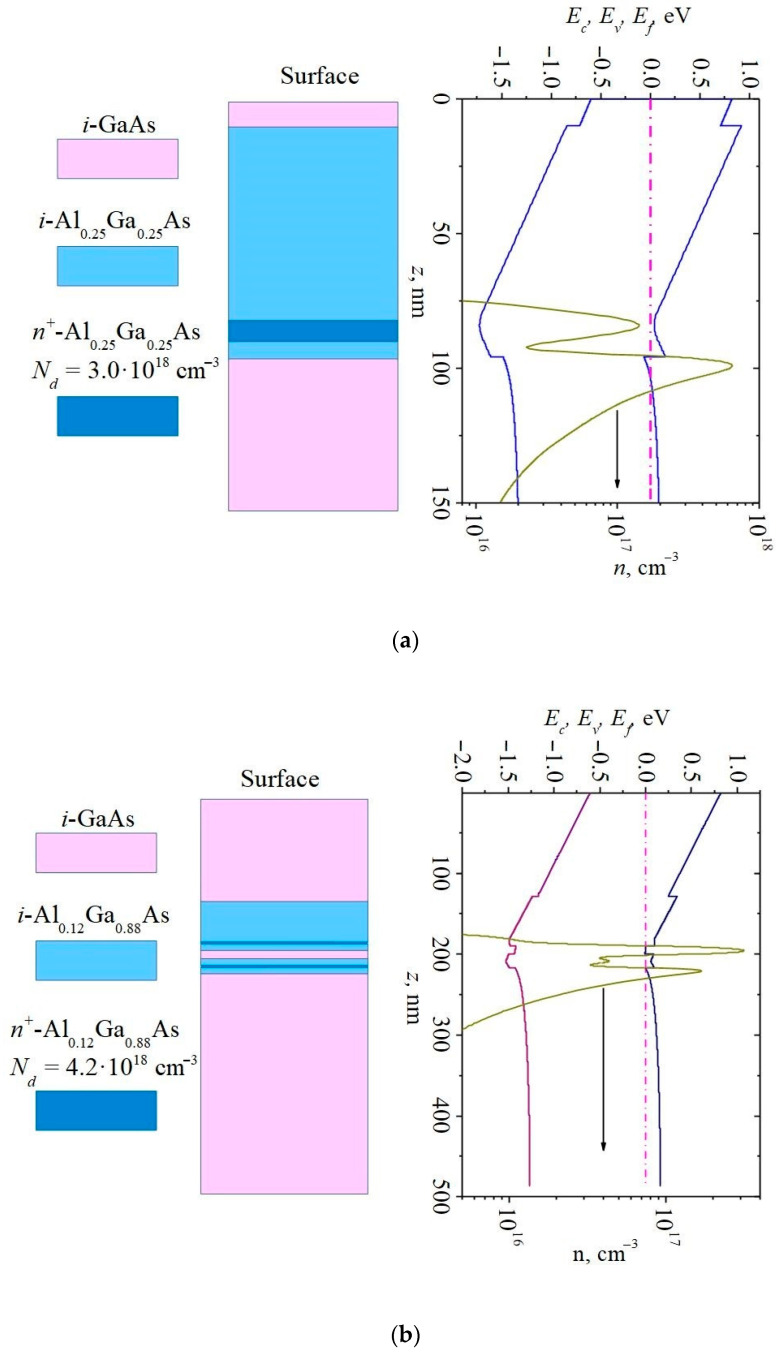

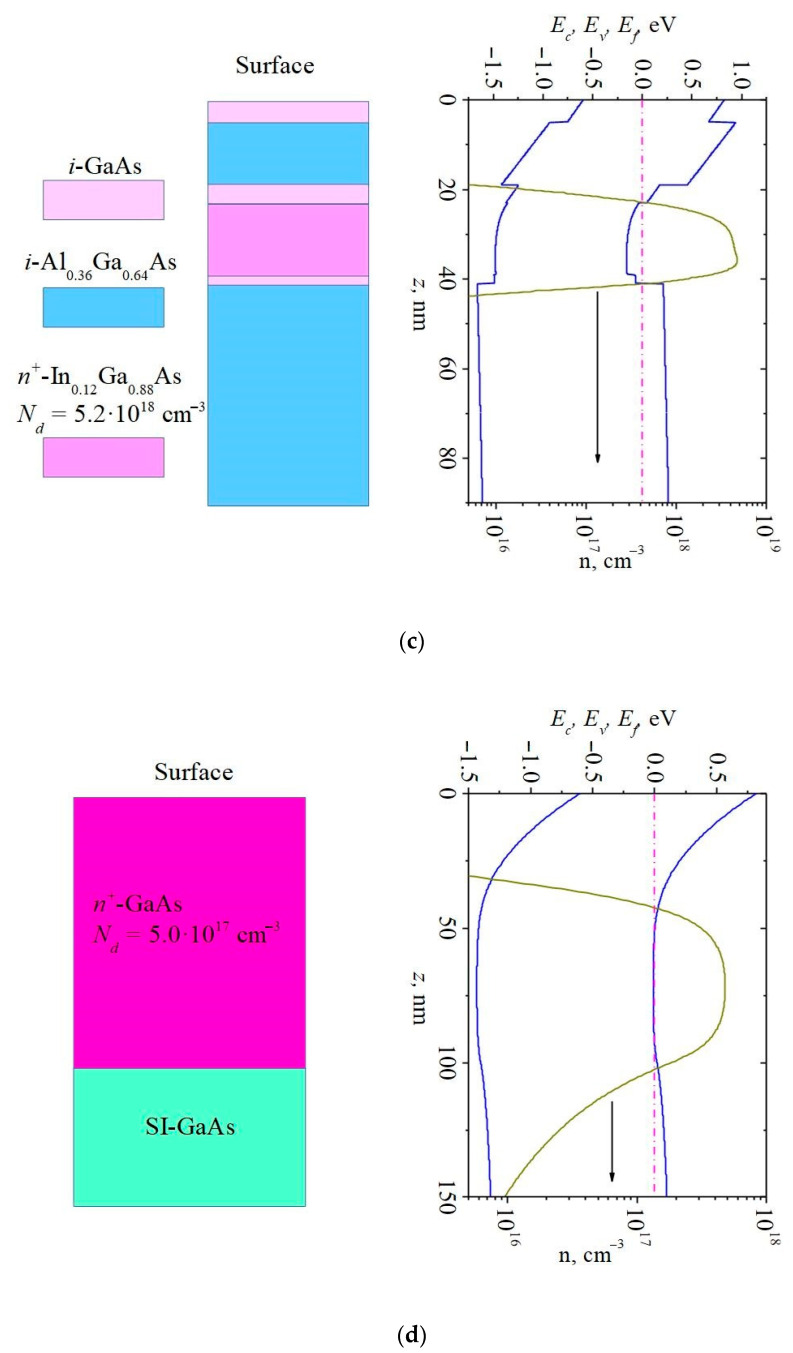

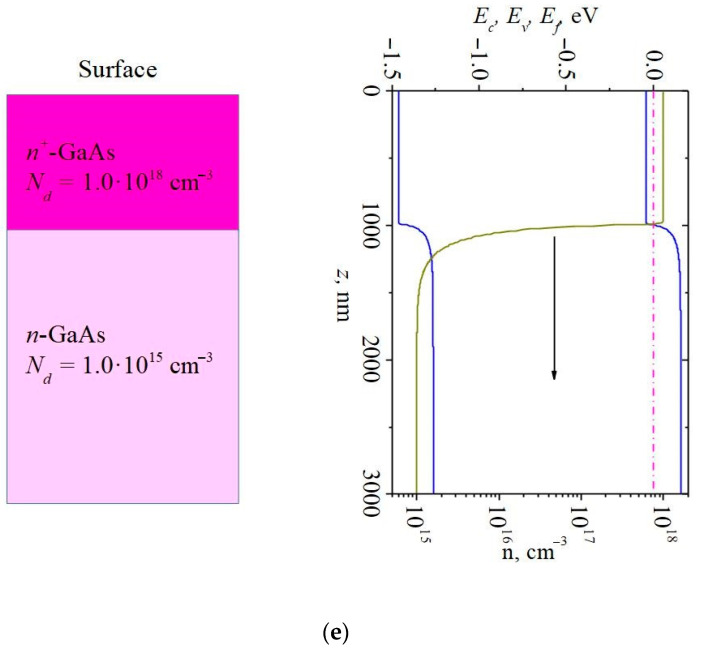
Cross-sections of the investigated semiconductor structures and their energy band diagrams with electron density distribution (in olive). (**a**) Selectively doped AlGaAs/GaAs structure with a triangular GaAs quantum well (TQW). (**b**) AlGaAs/GaAs structure with a rectangular GaAs quantum well (RQW). (**c**) GaAs/InGaAs structure with a rectangular InGaAs quantum well (InRQW). (**d**) Thin epitaxial heavily doped GaAs layer (THG). (**e**) Epitaxial structure with a thick lightly doped GaAs layer (TLG).

To determine the electron energy relaxation time values, four types of two-dimensional semiconductor structures were developed and fabricated: (*a*) selectively doped AlGaAs/GaAs structure with a triangular GaAs quantum well (TQW); (*b*) AlGaAs/GaAs structure with a rectangular GaAs quantum well (RQW); (*c*) GaAs/InGaAs structure with a rectangular InGaAs quantum well (InRQW); (*d*) thin epitaxial heavily doped GaAs layer (THG). An epitaxial structure containing a thick, lightly doped GaAs (TLG) layer was also investigated as a reference material. For clarity, the cross-sectional schematic view of the layered structures is presented in Figure A1 (Appendix A). Energy band diagrams and electron distribution in the structures were calculated by solving the Poisson equation and are also presented in Appendix A. The parameters of the structures are presented in Table 1.

Electron density and mobility in the active layer of the structures were derived from the Hall measurements. The experimentally measured density values agreed with the theoretical ones obtained by solving the Poisson equation within 25% precision.

**Figure 1:**
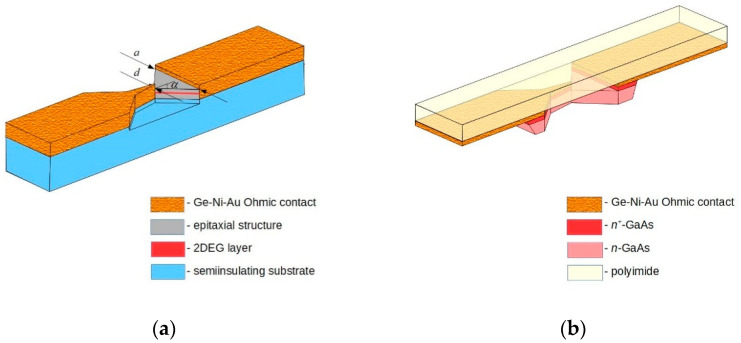
Schematic view of a planar, asymmetrically necked microwave diode on the base of (**a**) an epitaxial semiconductor layer situated on a semi-insulating substrate, and (**b**) bulky GaAs “glued” onto a polyimide film. Vertical dimensions are not to scale.

A schematic picture of the asymmetrically shaped planar MW diode on the base of the semiconductor epitaxial structure is presented in Figure 1a. This is the case of a diode on a semi-insulating substrate. The properties of such a diode can be investigated using probe stations both in DC and high-frequency regimes [35]. Therefore, it is possible to perform fast statistical evaluation of the diodes’ parameters, especially when it concerns high-frequency measurements. However, at present, the possibility to conduct high-frequency experiments by using the probe station is limited within the K_a_ frequency range. Therefore, research at higher frequencies requires placing the diodes into a waveguide. The “on semiconductor substrate” construction of the diode is not suitable for this purpose because a thick substrate being placed on the wide wall of a waveguide makes the diode penetrate sharply into the cavity of the high-frequency waveguide. For example, in the D frequency range (dimensions of the waveguide WR6 are 1.651 × 0.8255 mm^2^), a bulky 0.3-mm-thick microwave diode enters 36% into the waveguide cavity.

Therefore, another design of the MW diode is required at higher frequencies. A thin semiconductor structure with metallic contacts on an elastic dielectric polyimide film is the solution to this problem [33]. The most popular variant of the “filmy” MW diode is presented in Figure 1b.

**Figure 2:**
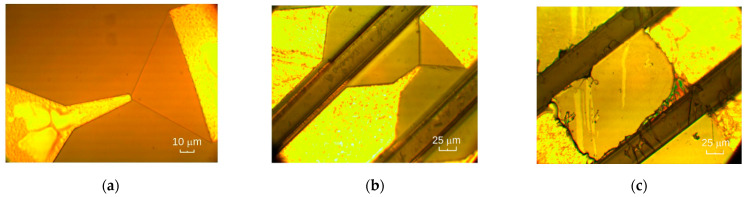
Micrographs of the planar, asymmetrically necked microwave diodes (**a**) on a semiconductor substrate; (**b**) on polyimide film (view from the polyimide film side); (**c**) on polyimide film (view from the side of metallic contacts).

The fabrication process of the asymmetrically shaped MW diodes began with wet chemical etching of the mesa structure using phosphorous acid solution. The etching depth was chosen to ensure the guaranteed confinement of the two-dimensional electron channels in the case of TQW, RQW, and InRQW diodes and it was equal to 100 nm, 250 nm, and 60 nm, respectively. Ohmic contacts of the diodes were fabricated by thermal evaporation of Ni:Au:Ge:Ni:Au metal layers of respective thicknesses of 5:200:100:75:100 nm onto a photo-resistive mask. The contact patterns were then formed by a lift-off technique. In the final stage, the contacts were annealed in a forming gas atmosphere according to the following heating regime: rise to 200 °C/hold at 200 °C/rise to 420 °C/hold at 420 °C/cooling stage, with respective time durations 10 s/60 s/10 s/60 s/150 s. The specific contact resistance and conductive layer sheet resistance were measured using differently spaced ohmic contacts on a rectangular semiconductor mesa [36]. The sheet resistance values of the formed semiconductor structures are presented in Table 1. The specific contact resistance was in the range of 0.06 to 0.14 Ω mm. At this stage of fabrication, the MW diodes were ready for the probe station investigation of their DC and high-frequency electrical properties. A micrograph of the asymmetrically necked microwave diode on a semiconductor substrate is presented in Figure 2a.

Micrographs of the MW diode positioned on a polyimide film are presented in Figure 2 as a view from the polyimide side (b) and as a view from the side of the metallic contacts (c). The procedure of fabrication began with the etching of deep limiting grooves around the diodes to a depth of ~5 μm from the top side of the layered structure. The purpose of these grooves was threefold: first, they separated the diodes from each other; second, their bottom marked the point where the semiconductor substrate needed to be thinned to (from the opposite side), and third, they marked the place where the diode’s mesa needed to be created during the stripping of the metallic contacts. The face side with the deep mesas was then covered with polyimide using a spin-on technique and curing at 250 °C for one hour in an ambient air atmosphere. The several-micrometer-thick polyimide film was strong enough and served as the mechanical diode’s support. Two wet chemical etching procedures finalized the process of fabrication of the planar diode: first, thinning the semiconductor substrate from its back until deep grooves appeared, and second, removing the rest of the semiconductor material from the ends of the metallic contacts.

The dependence of the voltage induced across the MW diodes on the incident microwave power was measured in the K_a_ frequency range using a Cascade Microtech high-frequency probe station. At higher frequencies, the detection properties were investigated by embedding separate MW diodes into a micro-strip-line [31]. The TWT generators operating in the W and D frequency bands were used as sources of microwave radiation. The radiation at *f* = 300 GHz frequency was generated by taking the second harmonic of the MW signal of *f* = 150 GHz using a frequency multiplier from Virginia Diodes Inc. All the measurements were performed at room temperature. When needed, the samples were illuminated by white light photo lamp Eiko EKE21V150W (color temperature 3240 K, intensity 50 W/m^2^).

## 3. Results and Discussion

**Figure 3:**
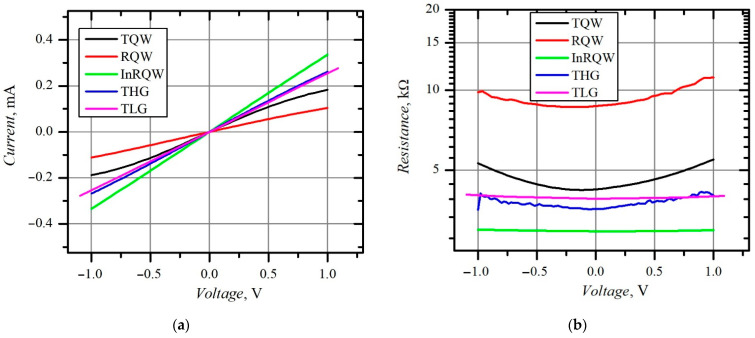
I-V characteristics (**a**) and dependence of electrical resistance on bias voltage (**b**) of different planar MW diodes; all measured in the dark.

At the first site, all the planar, asymmetrically shaped MW diodes demonstrated almost linear I-V characteristics within the ±1 V voltage range (see Figure 3a). These characteristics were measured in the dark. The nonlinearity of the I-V characteristics is evidenced by the dependence of electrical resistance on voltage, presented in Figure 3b. The illumination of the diodes reduced their electrical resistance. The relative decrease in zero-voltage resistance, (*R_drk_* – *R_ill_*)/*R_drk_*, where *R_drk_* and *R_ill_* denote the electrical resistance of the diode in the dark and under illumination, respectively, was mostly pronounced in the case of the InRQW diode and amounted to 18%. In the case of RQW, THG, TQW, and TLG diodes, the relative decrease was equal to 17%, 11.6%, 10.5%, and 6.8%, respectively. However, the experimentally measured electrical resistance values were higher than those calculated according to the relation

(6) $$R_{s0}=\frac{R_{sh}}{2}ln\frac{a}{d}.$$

Here, $R_{s0}$ is the geometrical electrical resistance of the asymmetrically necked semiconductor structure with *n-n^+^* junction described in Figure 1b. For example, the measured resistance value was from 90 to 120% higher than the theoretical one in the case of the TQW, RQW, and InRQW diodes with a 1-μm-wide neck. The greatest difference between the experimental ant theoretical values of the resistance was noticed in the case of the THG structure (up to ~200%). This difference became smaller with a wider neck (for example, the experimental values were 50 to 80% higher than the calculated ones when *d* = 3 μm). Such dependence on the neck width could be explained by a corresponding weaker influence of the specific contact resistance. However, the low value of the specific contact resistance rejects this version. For example, in the case of the THG diode with *d* = 1 μm, the contact resistance was 150 Ω, which amounted to only one tenth of the difference between the experimental and theoretical resistance values. The values of the sheet resistance and the specific contact resistance of the planar diodes are presented in Table 2.

Therefore, the reasons for the discrepancy between the theoretical and experimental results should be sought elsewhere. This difference can originate in the active region of the diode, i.e., in the narrowest part of it. The test structure prepared for the transmission line method measurements was a 100-μm-wide mesa with 100 × 100 μm contact islands separated at distances varying from 5 μm to 100 μm, and here, the experimentally measured electrical resistance coincided with the calculated geometrical resistance within the limits of error of the measurement. Thus, the physical background of the resistance increase should lie in semiconductor mesa-structures having micrometric dimensions. The flow of electrical current through the neck of the asymmetrically shaped semiconductor structure can be hindered by various surface recombination centers situated in the neck around the current flow channel, as was observed in an asymmetrically necked Si sample [37]. This presumption is supported by the fact that the illumination with the photo lamp diminishes the difference between the experimental and calculated resistances of the structures by 20 to 30%.

The asymmetry of the I-V characteristic of the asymmetrically necked planar semiconductor structure should satisfy Equation (4). First, the polarity of the I-V asymmetry should match the electrical current flow through the asymmetrically necked *n-n^+^* structure, i.e., the forward current should be assumed in the case when the negative potential is applied to the narrower part of the structure (“minus” to the *n^+^* region). The I-V asymmetry of the TQW, InRQW, and TLG diodes had right polarity in the dark, while the asymmetry of the RQW and THG structures had opposite polarity. When the structures were illuminated, only the TQW diode’s I-V asymmetry changed its polarity. Considering the I-V asymmetries of proper polarity, we calculated the electron energy relaxation time *τ_ε_* according to Equation (4). Table 3 summarizes the electron energy relaxation time values derived in the dark and under the white light illumination.

The value of electron energy relaxation time derived for the bulky GaAs asymmetrically necked TLG structure correlates with the *τ_ε_* = 0.60 ps value found from the I-V characteristic of the point-contact *n-n^+^*-GaAs diode [26] and with *τ_ε_* = 0.65 ps estimated from submillimeter conductivity measurements [38]. Application of the above method to find *τ_ε_* in cases of RQW and THG structures is questionable because their I-V characteristics do not match the typical characteristics of the asymmetrically necked semiconductor structure with *n-n^+^* junction and proper ohmic contacts. The comparatively higher *τ_ε_* value of the TQW structure pertains to the results obtained in quantum wells and bulk GaAs [29]. The electron energy relaxation time in the InRQW structure falls into the 0.5 to 1.7 ps range declared in ref. [22], where it was found in InGaAs quantum wells by means of microwave noise measurements. White light illumination of the InRQW and TLG structures reduced the asymmetry of their I-V characteristics, which resulted in an increased hot electron cooling rate.

The data presented in Table 3 cannot be treated unambiguously. Therefore, we searched for the electron energy relaxation time by using Equation (5), embracing the nonlinearity coefficient *β* of an I-V characteristic. Table 4 shows *β* values and the corresponding electron energy relaxation times for different planar MW diodes. The nonlinearity coefficient *β* was calculated using the following formula approximated to the forward branch of the experimental I-V characteristic,

(7) $$I=en_{s}d\mu_{0}\left(1-\beta E^{2}\right)E,$$

where *n_s_* is the electron surface density (cm^−2^), and *E* stands for the electric field strength in the narrowest part of the *n-*region of the asymmetrically necked semiconductor structure, and it is found as

(8) $$E=\frac{UR_{sh}}{R\left(U\right)d},$$

where *R*(*U*) is the electrical resistance of the planar microwave diode at an applied voltage *U*.

Comparison of the RQW, InRQW, and TLG data presented in Table 3 and Table 4 inspires one to conclude that the model of Equation (5) “nonlinearity of I-V characteristic” gives *τ_ε_* values two times lower than the model of Equation (4) “asymmetry of the I-V characteristic”. However, in both these cases, the same effect of light on electron energy relaxation time is observed: *τ_ε_* becomes slightly shorter when a diode is illuminated. It is worth noting that index *s* substantially influences the value of *τ_ε_* derived in any of these models. The parameter *s* is determined by the predominant electron scattering mechanism in a semiconductor. In our evaluations, we chose the prevalent scattering on polar optical phonons with *s* = 1 [34].

**Figure 4:**
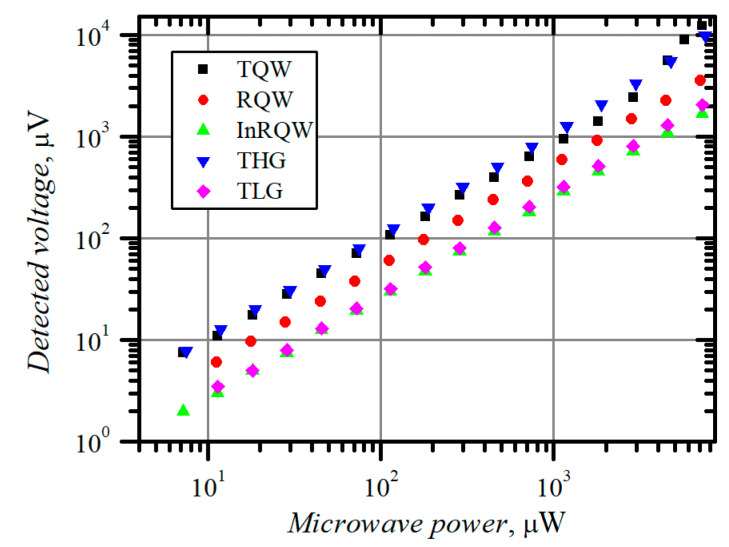
Voltage–power characteristics of planar, asymmetrically shaped MW diodes measured at *f* = 30 GHz in the dark.

**Figure 5:**
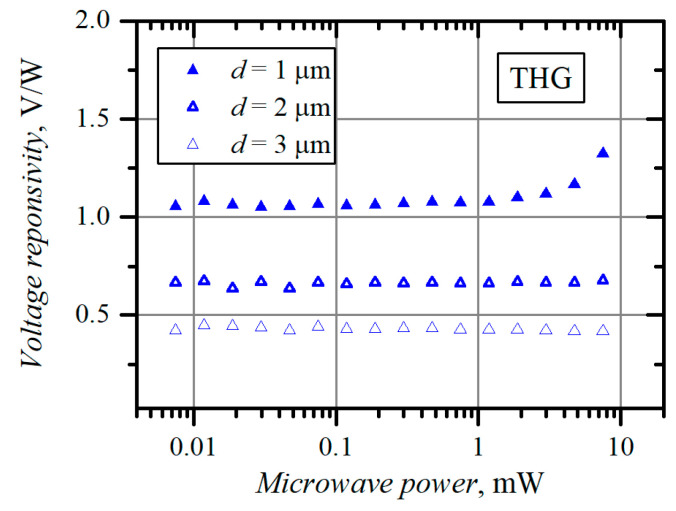
Dependence of voltage responsivity of the THG planar, asymmetrically necked MW diodes with different widths *d* of the neck. Microwave frequency is *f* = 30 GHz.

The ambiguity of *τ_ε_* values declared in Table 3 and Table 4 motivates us to look for other methods for finding the electron energy relaxation time. One of them is based on carrier heating by microwave radiation. The dependence of the voltage detected across the planar asymmetrically necked MW diodes, i.e., voltage–power (V-W) characteristic, measured at *f* = 30 GHz frequency in the dark using a probe station, is presented in Figure 4. The width of the neck of the diodes, except the TLG, was *d* = 1 μm. The width *d* and the thickness of the TLG structure were 9 μm and 2.6 μm, respectively. The polarity of the voltage detected across all the diodes corresponded to that of the hot electron electromotive force, showing positive potential arising on the contact of the more narrowed side of the structure (left side of the diode in Figure 1). RQW, InRQW, and TLG diodes show linear dependence of the voltage on microwave power in the measured microwave power range. In the case of TQW and THG diodes, the character of the dependence changes from linear to supper-linear at microwave power exceeding 1 mW. It is known that the voltage responsivity of *n*-*n^+^* junction-based MW diodes increases with the increase in electron energy relaxation time [39] and electron diffusion coefficient [40], as was evidenced in the case of the GaAs point-contact diode with *n*-*n^+^* junction [26]. The electric field strength in the neck of the RQW and TLG diodes is insufficient to initiate the increase in electron energy relaxation time and diffusion coefficient. Therefore, their V-W characteristics are linear. The absence of the superlinearity in the case of the InRQW structure can be explained by the different material of the conductive channel, which is not GaAs but InGaAs. Figure 5 depicts the dependence of voltage responsivity on microwave power for the THG planar, asymmetrically necked diodes with different widths *d* of the neck. The wider the neck, the lower the electric field in the narrowest part of the *n*-region of the structure. A weak increase in voltage responsivity can be observed in the case of *d* = 2 μm when the power exceeds 10 mW. It is worth noting that the illumination reduces the responsivity to some extent. However, unlike the case of the I-V asymmetry, the light does not influence the polarity of the detected voltage. The relative impact of the illumination on the voltage responsivity of all diodes is presented in Table 5.

The illumination-induced decrease in voltage responsivity most probably results from increased conductivity of the active layer of the structure (see Equation (1)). The most sensitive to illumination is the TQW sample because its active layer is closer to the exposed surface of the structure as compared, for example, with the RQW sample (see energy diagrams in Figure A1). The other diodes showed lower sensitivity to the illumination. The reason for this finding may lie in the fact that their conductive channels are formed by direct heavy doping and the conductive channels are comparatively further from the illuminated surface.

**Figure 6:**
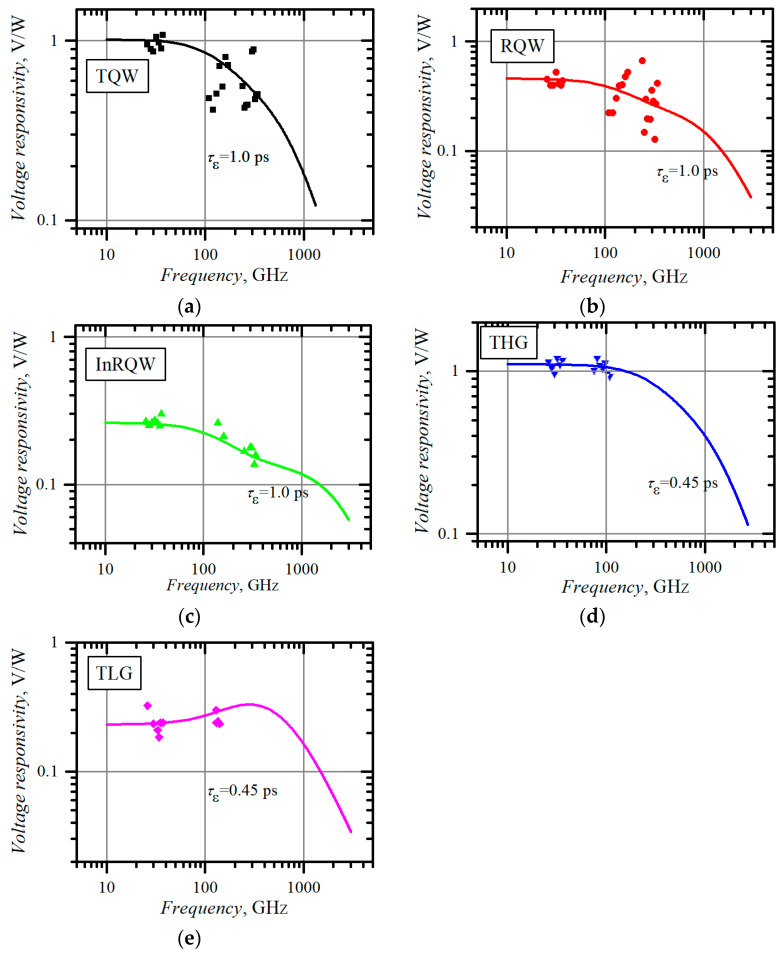
Experimental (points) and theoretical (lines) frequency dependence of the voltage responsivity of planar, asymmetrically necked MW diodes on the base of: (**a**) selectively doped AlGaAs/GaAs structure with a triangular quantum well, TQW; (**b**) AlGaAs/GaAs structure with a rectangular GaAs quantum well, RQW; (**c**) GaAs/InGaAs structure with a rectangular InGaAs quantum well, InRQW; (**d**) thin epitaxial heavily doped GaAs layer, THG; (**e**) epitaxial structure with a thick lightly doped GaAs layer, TLG.

The final method of our search of electron energy relaxation time is built on the voltage responsivity dependence on microwave frequency. The frequency dependences of the responsivity of the planar, asymmetrically necked MW diodes are presented in Figure 6.

All the measurements were carried out in the dark. Experimental dependences were approximated to Equation (1), with electron energy relaxation time *τ_ε_* and absorbed microwave power *P* taken as fitting parameters. Note that the approximation curve was insensitive to the electron scattering mechanism in the entire measured frequency range, i.e., the calculated result was the same with exponent *s* = 0 (scattering on ionized impurities) and with *s* = 1 (scattering by optical phonons). Table 6 summarizes electron energy relaxation time values in all the unilluminated planar, asymmetrically necked semiconductor structures obtained using the above-described methods of I-V asymmetry, I-V nonlinearity, and responsivity dependence on microwave frequency.

Average electron energy relaxation times are also presented in Table 6. The average *τ_ε-drk_* value of the THG structure leaves the assembly. Given that the THG sample failed to supply us with the electron energy relaxation time by using the I-V asymmetry method, one could argue that the estimates of this method should be questionable. Therefore, *τ_ε-drk_* values obtained using the responsivity-versus-frequency method should be treated as the most reliable since, in this case, the voltage responsivity is less dependent on the quality of the MW diode’s contacts and electron scattering mechanism in the active layer of the structure. In summary, the non-equilibrium energy of electrons in semiconductor structures with quantum wells relaxes more slowly than that in a bulk semiconductor, as was observed earlier by other authors [15,16,17].

## 4. Conclusions

The search for electron energy relaxation time in semiconductor structures with single quantum wells (2DEG structures) and bulk semiconductors by using analysis of their I-V characteristics and the dependence of their responsivity on microwave frequency allows us to conclude that

- estimation of *τ_ε_* from the I-V characteristic of the asymmetrically necked diode containing the *n-n^+^* junction is more sensitive to the quality of the ohmic contacts of the structure and to the electron scattering mechanism in a semiconductor as compared to the microwave method;
- the result obtained using the method of I-V characteristics is sensitive to illumination, and the observed reduction in electron energy relaxation time with illumination can be related to the light-caused decrease in the I-V asymmetry;
- the responsivity-versus-microwave frequency method gives more reliable values of carrier energy relaxation time because the experimental results are at least sensitive to the quality of contacts and to the electron scattering mechanism in a semiconductor;
- electron energy relaxation time in quantum well structures (2DEG structures) is two times longer than that in a bulk semiconductor, which correlates with the findings of other authors.

## Figures and Tables

**Table 1:** The geometrical and electrical parameters of the investigated semiconductor structures.

Structure	Shape and Thickness	Donor Doping Density *N_d_*, cm^−3^	Electron Density *n_s_*, cm^−2^	Electron Mobility *μ*_0_, cm^2^/(V·s)	Sheet Resistance *R_sh_*, Ω/ħ
**TQW**	Triangular QW 8.5 * nm	3.0 × 10^18^	1.2 × 10^12^	5500	940
**RQW**	Rectangular QW 10 nm	4.2 × 10^18^	1.0 × 10^12^	3260	2025
**InRQW**	Rectangular QW 16 nm	5.2 × 10^18^	6.0 × 10^12^	1540	745
**THG**	Thin layer 60 ** nm	5.0 × 10^17^	3.0 × 10^12^	2930	690
**TLG**	Thick layer 1000 nm	1.0 × 10^15^	1.0 × 10^15^ cm^−3^	6000	-

* Effective QW width *w* at the level of the lowest electron state. ** Effective thickness of the structure at the depth where electron density drops down *e* times.

**Table 2:** Sheet resistance *R_sh_* and specific contact resistance *ρ_c_* of the planar, asymmetrically necked diodes in dark and under illumination.

Structure	Illuminated		Dark	
	*R_sh_*, Ω/ħ	*ρ_c_*, Ω·mm	*R_sh_*, Ω/ħ	*ρ_c_*, Ω·mm
**TQW**	890	0.055	940	0.185
**RQW**	1910	0.074	2025	0.09
**InRQW**	700	0.11	745	0.14
**THG**	590	0.14	690	0.15

**Table 3:** Electron energy relaxation time *τ_ε-ill_* and *τ_ε-drk_* under illumination and in the dark, respectively, evaluated from I-V characteristics of various asymmetrically necked semiconductor structures [model of Equation (4)].

Structure	TQW	RQW	InRQW	THG	TLG
***τ_ε-ill_*, ps**	-	2.4	0.22	2.3	0.31
***τ_ε-drk_*, ps**	2.2	-	0.85	-	0.53

**Table 4:** Electron energy relaxation time *τ_ε-ill_* and *τ_ε-drk_* under illumination and in the dark, respectively, evaluated from corresponding I-V characteristic’s nonlinearity coefficients *β_ill_* and *β_drk_* of different asymmetrically necked semiconductor structures (model of Equation (5)).

Structure	*β_ill_* × 10^12^, m^2^/V^2^	*τ_ε-ill_*, ps	*β_drk_* × 10^12^, m^2^/V^2^	*τ_ε-drk_*, ps
**TQW**	−16.0	1.1	−17.3	1.2
**RQW**	−1.6	0.19	−3.5	0.41
**InRQW**	1.6	0.38	1.7	0.41
**THG**	−14.5	1.9	−15.0	1.9
**TLG**	−3.0	0.19	−3.5	0.22

**Table 5:** The relative change in voltage responsivity of different diodes as a result of their reaction to illumination. *S_drk_* is the responsivity in the dark, and *S_ill_* notes it under illumination.

Structure	TQW	RQW	InRQW	THG	TLG
**(*S_drk_ − S_ill_*)/*S_d_*, %**	40	20	12	3	8

**Table 6:** Electron energy relaxation time *τ_ε-drk_* evaluated using I-V asymmetry, I-V nonlinearity, and responsivity-versus-frequency methods in different planar, asymmetrically necked MW diodes.

Structure	*τ_ε-drk_*, ps			
	From I-V Asymmetry	From I-V Nonlinearity	From MW Frequency Dependence	Average
**TQW**	2.2	1.2	1.0	1.5
**RQW**	-	0.41	1.0	0.7
**InRQW**	0.85	0.41	1.0	0.75
**THG**	-	1.94	0.45	1.2
**TLG**	0.53	0.22	0.45	0.4

## Data Availability

Data sharing is not applicable to this article.
